# Effect of early stellate ganglion block in cerebral vasospasm after aneurysmal subarachnoid hemorrhage (BLOCK-CVS): study protocol for a randomized controlled trial

**DOI:** 10.1186/s13063-022-06867-9

**Published:** 2022-11-04

**Authors:** Longnian Jing, Youxuan Wu, Fa Liang, Minyu Jian, Yang Bai, Yunzhen Wang, Haiyang Liu, Anxin Wang, Xiaolin Chen, Ruquan Han

**Affiliations:** 1 Department of Anesthesiology, Fengtai District, Beijing Tiantan Hospital, Capital Medical University, No. 119, Southwest 4Th Ring Road, Beijing, People’s Republic of China 100070; 2 China National Clinical Research Center for Neurological Diseases, Beijing, People’s Republic of China 100070; 3 Department of Neurosurgery, Beijing Neurosurgical Institute, Beijing, People’s Republic of China 100070

**Keywords:** Stellate ganglion block, Cerebral vasospasm, Subarachnoid hemorrhage, Prevention, Effectiveness, Safety

## Abstract

**Introduction:**

Stellate ganglion block has been reported to expand cerebral vessels and alleviate vasospasm after aneurysmal subarachnoid hemorrhage. However, the causal relationship between early stellate ganglion block and cerebral vasospasm prevention has not yet been established. The purpose of this study was to explore the effectiveness and safety of early stellate ganglion block as a preventive treatment for cerebral vasospasm and delayed cerebral ischemia.

**Methods/design:**

This is a single-center, prospective, randomized, controlled, blinded endpoint assessment superiority trial. A total of 228 patients will be randomized within 48 h of aneurysmal subarachnoid hemorrhage onset in a 1:1 ratio into two groups, one group receiving an additional e-SGB and the other group receiving only a camouflaging action before anesthesia induction in the operating room. The primary outcome is the incidence of symptomatic vasospasm within 14 days after aSAH. Further safety and efficacy parameters include the incidence of radiographic vasospasm, new cerebral infarction, postoperative delirium, and complications up to 90 days after surgery; postoperative cerebral hemodynamics; Mini-Mental State Examination score; modified Rankin scale score; and all-cause mortality up to 90 days after surgery.

**Discussion:**

This is a randomized controlled trial to explore the effectiveness and safety of early stellate ganglion block as a preventive treatment to reduce cerebral vasospasm in patients with aneurysmal subarachnoid hemorrhage. If the results are positive, it may provide a new direction for the prevention and treatment of cerebral vasospasm and delayed cerebral ischemia.

**Trial registration:**

The study was registered on Clincaltrials.gov on December 13, 2020 (NCT04691271).

**Supplementary Information:**

The online version contains supplementary material available at 10.1186/s13063-022-06867-9.

## Background

Aneurysmal subarachnoid hemorrhage (aSAH) is a serious life-threatening disease caused by blood flowing into the subarachnoid space after a ruptured intracranial aneurysm. The worldwide incidence of aSAH is approximately 9.1/100,000 people/year [1–3]. In the past 30 years, surgical treatment of ruptured aneurysms has greatly reduced the risk of aneurysm rerupture, and global case mortality has been reduced by 17 to 50% [4–7]. Substantial progress has been made in the treatment of ruptured aneurysms, but little progress has been made in the prevention and treatment of delayed cerebral ischemia (DCI) associated with cerebral vasospasm (CVS). Consequently, DCI after aSAH, especially that associated with CVS, remains a major cause of death and long-term disability, and the disability rate is greater than one-third [4, 5].

CVS is considered a vital underlying mechanism, although the formation mechanism of DCI is not clear. Studies have shown that the incidence of CVS is more than 70%, of which 20–30% of cases can cause DCI [5–8]. Increasing evidence supports that the inflammatory cascade and early brain injury (EBI), which continue and cause various brain pathophysiologies within the first 72 h after bleeding, may be important factors in the formation of CVS and DCI [2, 9–12]. Of these, neuroinflammation is related to almost all pathological processes contributing to CVS or DCI. Thus, it can be understood why all pharmacologic measures based only on vasodilatory properties demonstrate limited efficacy, and even nimodipine is believed to exert its beneficial effect by neuroprotective mechanisms rather than its vasodilatory properties [2, 13]. Blocking inflammation-mediated processes in an early and timely manner may be a more therapeutically effective way to reduce CVS or DCI and improve clinical outcome. Despite years of effort, some tertiary medical centers still report that the incidence of symptomatic or radiographic vasospasm is unacceptably high, at 20–50% or as high as 70% [14–20], respectively. Thus, the prevention and treatment of CVS and DCI is still a major clinical problem.

The stellate ganglion (SG) belongs to the sympathetic ganglion, formed by a fusion of the inferior cervical and first thoracic sympathetic ganglia. It can modulate the immune response, diminish inflammation, and improve cerebral perfusion, as confirmed by numerous studies [21–29]. Current studies have tried stellate ganglion block (SGB) in aSAH patients with diagnosed CVS and observed beneficial outcomes [23, 29–33]. However, early prevention of CVS seems to be more meaningful than treatment of progressed CVS. The modulating effect of SGB on the immune system and its improvement of cerebral perfusion to reduce EBI provide the possibility for the prevention of CVS and DCI. However, the causal relationship between early SGB (e-SGB) and CVS prevention has not yet been established. We designed this study to explore the effectiveness and safety of e-SGB as a means of CVS prevention. We hypothesize that early use of SGB is safe and effective in reducing the incidence of CVS. The results will provide basic evidence for prophylactic e-SGB for CVS and even DCI.

## Methods

### Objective

This trial aims to explore the effectiveness and safety of early stellate ganglion block as a preventive treatment for cerebral vasospasm and delayed cerebral ischemia by observing the incidence of symptomatic vasospasm within 14 days after aSAH.

### Trial design

**Fig. 1:**
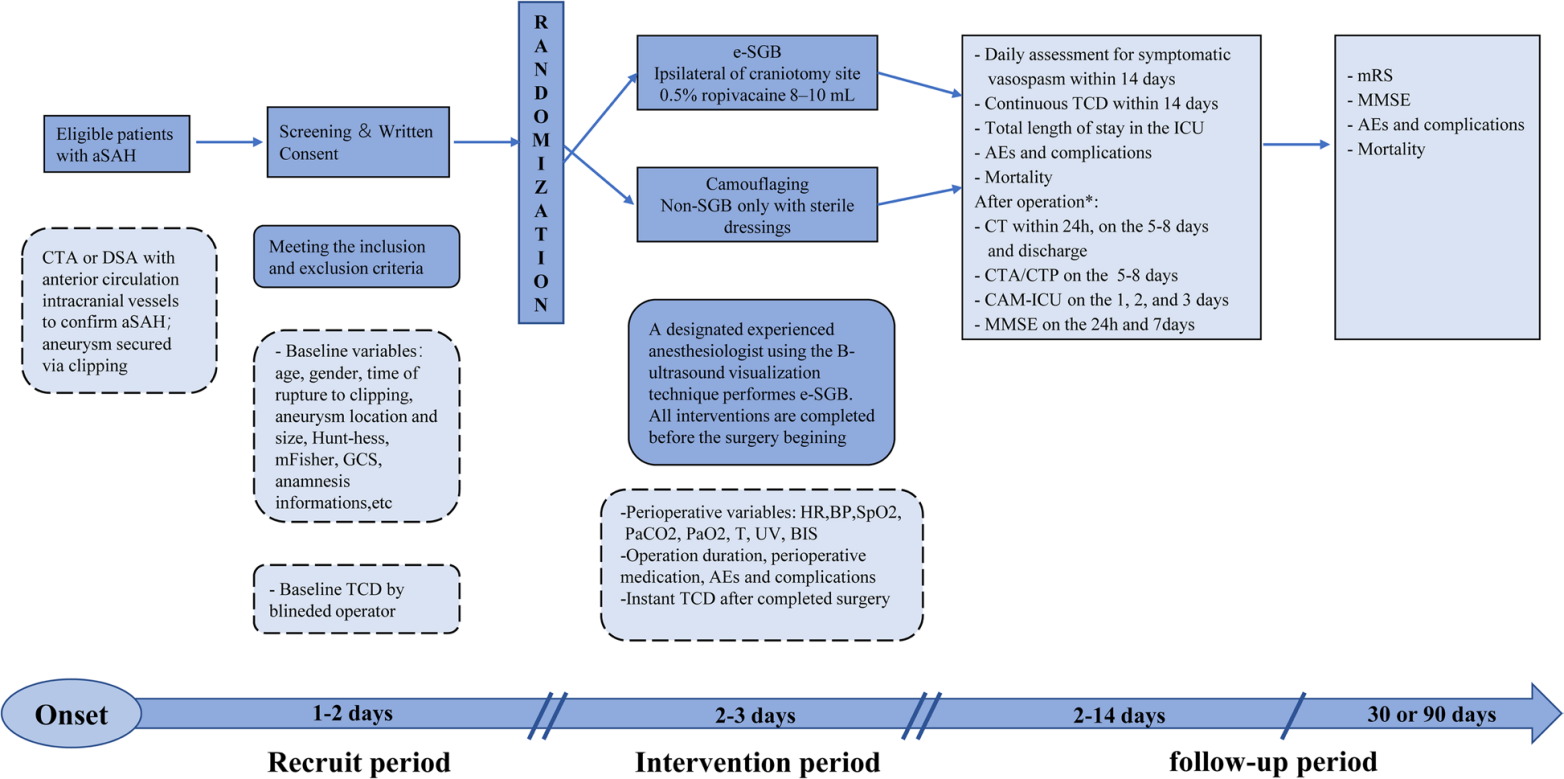
Study implementation flow chart. The follow-up was completed by a special blinded team, including neurologists, radiologists, nurses, and graduate students. Asterisk symbol (*) indicates that the following time node starts from this moment. Hunt-hess, Hunt and Hess grading; mFisher, modified Fisher grading; GCS, Glasgow Outcome Scale; TCD, transcranial Doppler ultrasound; SGB, stellate ganglion block; HR, heart rate; BP, blood pressure; SpO_2_, pulse oxygen saturation; PaCO_2_, arterial partial pressure of carbon dioxide; EtCO2, end-tidal carbon dioxide; T, temperature; UV, urine volume. BIS, bispectral index; ICU, intensive care unit; AEs, adverse events; CAM-ICU, Confusion Assessment Method for the Intensive Care Unit; MMSE, Mini-Mental State Examination; mRS, modified Rankin Scale

This trial is a prospective, randomized, open-label, blinded endpoint assessment superiority study that is in line with the Standard Protocol Items: Recommendations for Interventional Trials (SPIRIT 2013). Its two parallel arms do not cross each other, and the random distribution probability is the same. A total of 228 aSAH patients will be randomized within 48 h of symptom onset to an intervention group receiving an additional e-SGB before aneurysm clipping or to a control group undergoing only a routine treatment procedure. The end of follow-up will be 90 days after surgery. The schedule of events for the enrollment, interventions, and assessments of participants is shown in Fig. 1 and Table 1.

**Table 1:** SPIRIT flow diagram

Time point (from onset)	Operation			Post-operation follow-up				
	**Pre**	**Dur**	**Aft**	**Day**	**Day**	**Day**	**Discharge**	**Day 30 or**
	**Day 1–2**			**2–6**	**7–10**	**10–14**		**Day 90**
**Enrollment**								
Eligibility screen	✖							
Recruitment	✖							
Consent	✖							
Randomization and allocation	✖							
**Interventions**								
SGB	✖							
Camouflage action (non-SGB)	✖							
Block-related information	✖							
**Assessments**								
Baseline variables	✖							
Neurological examination	✖		✖	✖	✖	✖	✖	
Physiological parameters	✖	✖	✖	✖	✖	✖	✖	
MMSE	✖		✖		✖		✖	✖
CAM-ICU	✖		✖	✖				
mRS							✖	✖
TCD	✖		✖	✖	✖	✖		
CT	✖		✖		✖		✖	
CTA/CTP	✖				✖			
Complications		✖	✖	✖	✖	✖	✖	✖
All adverse events		✖	✖	✖	✖	✖		
Length of stay							✖	
All-cause mortality							✖	✖

*SGB* Stellate ganglion block, *TCD* Transcranial Doppler, *MMSE* Mini-Mental State Examination, *CAM-ICU* Confusion Assessment Method for the Intensive Care Unit, *mRS* modified Rankin scale, *CT* Computed tomography, *CTA* Computed tomography angiography, *CTP* Computed tomography perfusion imaging

### Location and setting

This trial intends to recruit subjects from Beijing Tiantan Hospital, Capital Medical University, from July 1, 2021, to December 30, 2023.

### Patient population

#### Inclusion criteria

Patients with aSAH diagnosed by imaging (CTA or DSA) and scheduled for surgical intervention within the expected time period (48 h) will be assessed for recruitment. Those with an onset more than 48 h prior to assessment will not be included because CVS can occur on the third day after onset. The inclusion criteria were as follows: (i) 18 years ≤ age ≤ 65 years, (ii) planned aneurysm clipping, (iii) preoperative Hunt-Hess grade 1–3, and (iv) signed informed consent.

#### Exclusion criteria

Patients meeting at least one of the following exclusion criteria will be considered ineligible to participate: (i) ASA physical status > III; (ii) posterior circulation aneurysm, ophthalmic aneurysm, or internal carotid aneurysm; (iii) multiple aneurysms; (iv) coagulation dysfunction; (v) trauma and local infection in the nerve block area; (vi) neck anatomic structural changes (caused by radiotherapy, chemotherapy or surgery); (vii) poor temporal window signal revealed by preoperative transcranial Doppler; (vii) middle cerebral artery stenosis or infarction found by preoperative imaging; (ix) allergy to known local anesthetics; or (x) pregnant or lactating women or those unwilling to sign the informed consent form.

### Recruitment and consent

At least two designated anesthesiologists will participate in the recruitment, randomization, and intervention. One is the attending physician, mainly responsible for the randomization, intervention, and clinical anesthesia in the operating room, and the other is a resident physician, mainly responsible for research recruitment and baseline data collection at the preoperative visit before the patient enters the operating room. The patients will be preliminarily screened by an independent and experienced staff member (Chief Neurosurgery Resident for Consultation) according to the inclusion and exclusion criteria. He will notify the designated anesthesiologists in advance if the patient meets the enrollment criteria, and then the designated resident anesthesiologists will go to the emergency room for preoperative visits, including rechecking the eligibility and obtaining informed consent. Reasons why eligible patients are not recruited for the trial will be documented. The family members will be informed of the relevant information, including detailed investigational treatment and potential risks and benefits, after the patient meets the inclusion/exclusion criteria. In addition, the patients can be formally recruited only after the staff obtains the informed consent of the patients or their next of kin.

### Randomization and blinding

Randomization will occur on the date of craniotomy aneurysm clipping. Permuted block will be used and stratified by the culprit vessel sites, including the middle cerebral artery, anterior cerebral artery, anterior communicating artery, and posterior communicating artery. The ophthalmic artery, internal carotid artery, and posterior circulation arteries will not be included in this study. The randomized digital table was previously computer (STATA 17) generated by dedicated independent staff and loaded into a web-based database. The allocation information will be obtained by the attending anesthesiologist through mobile phone software after the patient enters the operating room. Patients will be randomly allocated to receive either e-SGB or camouflaging in a 1 to 1 ratio. The endpoint assessors, data statisticians, and postoperative treatment team members will be blinded to the treatment group allocation, but the anesthesiologist and neurosurgeons will not be blinded, as they need to participate in the safe administration of related patient care. Patients will be unblinded only if they experience serious adverse events requiring additional visits.

### Intervention

**Fig. 2:**
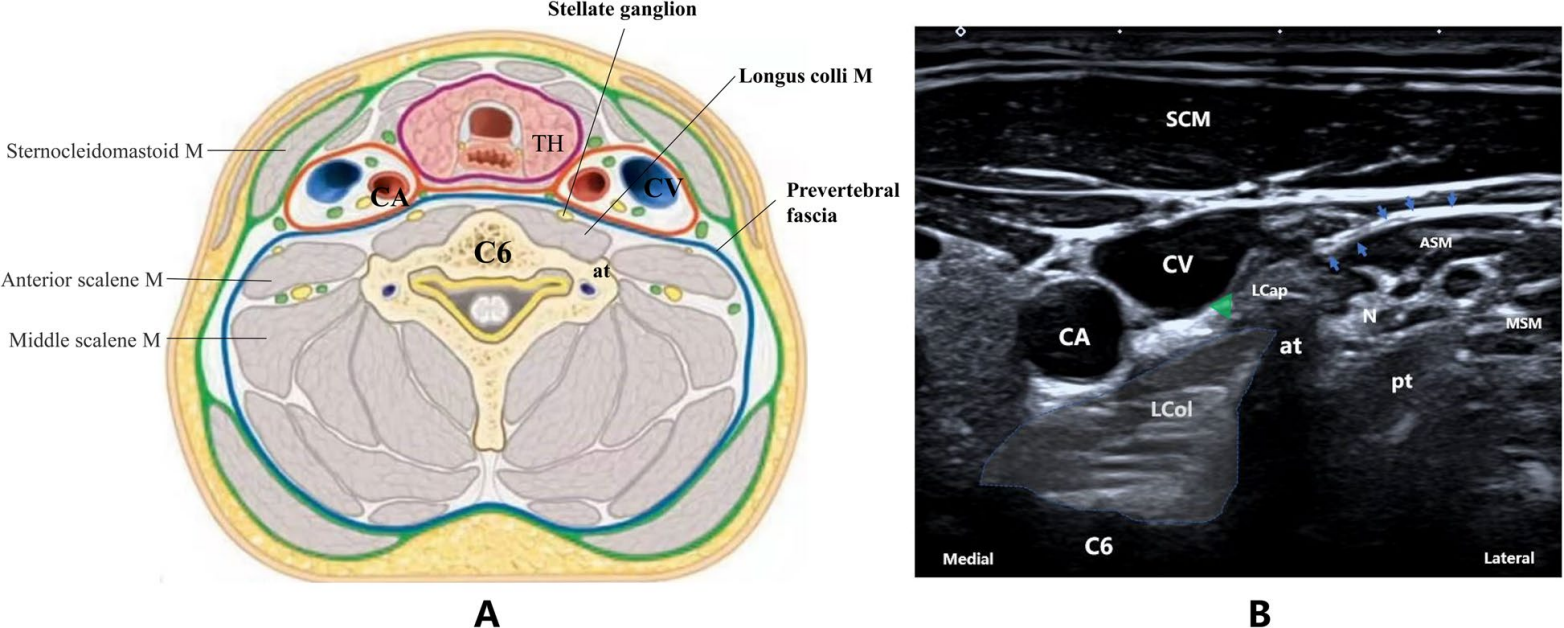
Schematic diagram of stellate ganglion block under ultrasound guidance. After routine disinfection, 0.5% ropivacaine 8–10 mL will be injected into the surface of the longus colli muscle on the medial side of the prevertebral fascia at the level of the C6 anterior tubercle, and then the puncture point will be covered with sterile dressings. **B** The ultrasonic image of the cross-section of the sixth cervical segment: the blue arrow indicates the prevertebral fascia; the blue dotted line area is the longus colli muscle, and its lateral highlight area is the distribution of the stellate ganglion; and the green triangle indicates the drug injection site (the inner side of the prevertebral fascia), in which the stellate ganglion can be effectively blocked through the diffusion of the drug solution under the fascia and without nerve injury. C6, the sixth cervical segment; CA, carotid artery; CV, jugular vein; TH, thyroid; at, anterior tubercle; M, muscle; SCM, sternocleidomastoid muscle; LCol, longus colli muscle; LCap, longus capitis muscle; N, nerve from cervical plexus; pt, posterior tubercle. ASM, anterior scalene muscle; MSM, middle scalene muscle

Randomized patients will receive an additional e-SGB or a “camouflage” operation before anesthesia induction and then receive standard care after the operation. In the intervention group, e-SGB will be performed by a designated experienced anesthesiologist using the B-ultrasound visualization technique. The intervention site will be the ipsilateral side of the planned craniotomy site. After routine disinfection, 8–10 mL of 0.5% ropivacaine will be injected into the surface of the longus colli muscle on the medial side of the prevertebral fascia at the level of the C6 anterior tubercle (Fig. 2), and the puncture point will be covered with sterile dressings. The success criterion of e-SGB is Horner's syndrome, which is characterized by miosis, ptosis, enophthalmos, conjunctival hyperemia, and facial redness without sweating. The patient will be monitored for at least 30 min before anesthesia induction to rule out any related complications after SGB. Patients with failed e-SGB will be excluded. In the control group, a blank control was adopted to reduce unnecessary invasive operations. Patients will not undergo any sham procedures and will only be prospectively visited at a specified time point. However, they need to undergo the “camouflage” operation to ensure the smooth implementation of the blinded endpoint evaluation. The anesthesiologist will cover the corresponding part of the patient with sterile dressings to confuse the follow-up, without any puncture. In this way, the follow-up staff would not be aware of the grouping of the participants by the puncture point. All patients will be admitted to the ICU after the operation and then receive the standard of care.

### SGB-related complications

Complications related to SGB can be greatly reduced by ultrasound-guided procedures. Although the incidence is low, experienced anesthesiologists will be involved, and SGB-related complications will be regarded as safety indicators in this study. Patients who present suspicious symptoms within 30 min after e-SGB will be treated immediately, and a preliminary diagnosis will be graded by the anesthesiologist (Table 2). According to the patient’s condition, the anesthesiologist and the neurosurgeon will decide jointly whether to suspend the operation or implement further treatment. For serious complications (grading criteria ≥ 3), additional follow-up may be required to ensure patient safety; in those cases, the patient will be unblinded, but we will continue follow-up outcomes unless the patient or their families request withdrawal from this study. Serious complications, such as subarachnoid block, will be immediately reported to the ethics committee by the PI, and an emergency data monitoring committee meeting will be initiated for consultation. The possible complications of SGB and the time of observation for each are as follows:

- Block or injury of nerve: recurrent laryngeal, phrenic, brachial plexus, and vagus nerves (30 min after e-SGB and during hospitalization)
- Injury of the artery: internal carotid and vertebral arteries (30 min after e-SGB)
- Local anesthetic poisoning (30 min after e-SGB)
- Subarachnoid block (30 min after e-SGB)
- Pneumothorax and hemothorax (30 min after e-SGB)
- Infection and hematoma at the puncture site (during hospitalization)

**Table 2:** Classification of complications

Grade	Definition
Grade 1	No need for drugs or surgery, endoscopy, or intervention
Grade 2	Requiring some treatment, including blood transfusions and parenteral nutrition
Grade 3	Requiring surgical, endoscopic, or radiological intervention
3a	Intervention not under general anesthesia
3b	Intervention under general anesthesia
Grade 4	Severe life-threatening complications need to be transferred to the intensive care unit or special care unit
4a	Single-organ dysfunction
4b	Multiorgan dysfunction
Grade 5	Resulting in death

### Standardized anesthetic management

All recruited patients will receive unified perioperative anesthesia management (see Supplement Part 1 for details). Anesthesia with propofol, sufentanil, and remifentanil will be administered to maintain the target BIS values between 40 and 60. The fluctuations in SBP will be maintained within 20% of the baseline. For preoperative SBP > 160 mmHg, especially in patients with acute hypertension (SBP > 200 mmHg), SBP should be actively controlled to less than 160 mmHg regardless of the 20% range limit. During the perioperative period, it is necessary to maintain normal blood volume and avoid hypervolemia and hemodilution. Mechanical ventilation will avoid hypoxemia, hypercapnia, and hypocapnia. Since low hemoglobin may be detrimental to brain tissue oxygen supply, Hb > 10 g/dl or HCT > 30% should be maintained as much as possible. Blood glucose and electrolytes will be closely monitored and maintained in the normal ranges. Cerebral salt wasting syndrome will be treated with hydrocortisone or 3% saline. Finally, short-term prophylactic antiepileptic therapy is permitted in our center.

### Patient management after surgery

All patients will be admitted to the ICU and treated with a standard treatment protocol (see Supplement Part 2 for details) that includes intensive care monitoring, maintaining normotension (SBP 140 mmHg-220 mmHg) and normovolemia (positive fluid balance > 500 ml/day), decreasing intracranial pressure (ICP) and nimodipine therapy. Continuous intravenous infusion of nimodipine 0.8–2.0 mg/h is preferred at our center. Blood pressure will be maintained within a high threshold range in all patients, but we will not actively use induced hypertension to alleviate the ischemic symptoms. Likewise, hypervolemia and hemodilution will not apply in our center. Norepinephrine is the first choice as a vasopressor. Patients with severe cerebral edema or ICP greater than 20 mmHg will be treated with 3% NaCl saline or mannitol, preferably hypertonic saline, due to its minimal damage to the kidney. For patients with severe neurological deficits that cannot be explained by rebleeding, hydrocephalus, infection, surgical complications, etc., emergency DSA angiography will be performed, and intra-arterial vasodilators (Fasudil) or transluminal balloon angioplasty (TBA) will be given if necessary.

### Measurements

The endpoint assessors, who are only responsible for postoperative follow-up, will regularly visit all patients at the planned time points (Table 1). The nurse will check the patients every 4 h. At 10:00 AM every day, the attending neurologist and nurse will evaluate whether the patient experienced symptomatic vasospasm in the last 24 h and record it in the case report form. All patients will receive brain imaging, including CT, CTA, and CTP examination, at least twice, before the operation and 5–8 days after the operation. Postoperative days 5–8 correspond to the peak period of CVS appearance 7–10 days after aSAH. Imaging aims to assess CTA vasospasm, cerebral perfusion, new hemorrhage (if present), and infarction. CT needs to be examined an additional two times, one within 24 h after the operation to assess whether new cerebral infarction or hemorrhage is caused by surgery and one at discharge to assess the occurrence of new cerebral infarction. The assessments will be completed mainly by an independent radiologist. All TCD monitoring will be performed by an experienced and specialized follower on the day before surgery (T0) and on the immediate (T1), 1st (T2), 2nd (T3), 3rd (T4), 5th (T5), 7th (T6), and 9th (T7) days after the operation. Delirium will be documented using the CAM-ICU during the first 3 days after the operation. Additionally, cognitive performance will be assessed at baseline and at 24 h, 7 days, 30 days, and 90 days after surgery using the Mini-Mental State Examination (MMSE). The modified Rankin scale (mRS) will be used to assess patient disability at discharge and at 90 days. The incidence of complications, including myocardial infarction, postoperative rebleeding, moderate and severe brain edema, pulmonary embolism, deep venous thrombosis, SGB-related complications, and unexpected adverse events, will also be recorded. All baseline and intraoperative data will be recorded by the resident anesthesiologists and the attending anesthesiologists who are responsible for recruitment and intervention.

### Primary outcome

The primary efficacy outcome is the incidence of symptomatic vasospasm within 14 days after aSAH, which is defined as new focal or global neurological dysfunction or a decrease in the Glasgow coma score by more than two points, with angiographic vasospasm on TCD or CTA [34–36]. Symptoms must not be explainable by causes such as primary bleeding, rebleeding, hydrocephalus, fever, infection, electrolyte or metabolic disorders, and surgical complications.

### Secondary outcomes

The secondary outcomes in the BLOCK-CVS trial include the following:

- Incidence of TCD vasospasm within 14 days after aSAH [34].
- Incidence of CTA vasospasm on days 5–8 after the operation [34].
- Incidence of abnormal perfusion monitored by CTP on days 5–8 after the operation
- Incidence of new cerebral infarction observed on days 5–8 after the operation and at discharge
- Continuous cerebral blood flow velocity monitored by TCD on the day before surgery (T0) and on the immediate (T1), 1st (T2), 2nd (T3), 3rd (T4), 5th (T5), 7th (T6), and 9th (T7) days after the operation
- The Mini-Mental State Examination (MMSE) score was assessed at 24 h, 7 days, 30 days, and 90 days after the operation
- Incidence of delirium as measured by CAM within 3 days after the operation
- Total length of stay in the intensive care unit and hospital
- Modified Rankin Scale (mRS) score at discharge, 30 days, and 90 days
- All-cause mortality up to 90 days
- Incidence of complications up to 90 days

See Supplement Part 3 for detailed definitions of all observed indicators.

### Sample size calculation

At our institution, we have observed a symptomatic vasospasm rate of approximately 45%. The cure rate of SGB in the treatment of progressive CVS was reported as 39% in another trial [31]. Therefore, we hypothesize a positive effect size of 40% for e-SGB compared with the baseline rate. Finally, using PASS 15, a total of 228 patients were needed to detect the difference (27% in the treatment arm vs. 45% in the control arm) in the primary outcome with 80% power and a two-sided alpha of 0.05, allowing for a 5% loss to follow-up. An interim analysis will be performed when 50% of the required sample size is recruited.

### Statistical analysis

All efficacy and safety analyses will be analyzed on both a per-protocol and intention-to-treat basis. The intent-to-treat (ITT) population will comprise all subjects randomized to receive e-SGB and with the completed assessment of symptomatic CVS. The per-protocol (PP) population will consist of all subjects in the ITT population not identified as protocol violators (i.e., no obvious Horner’s syndrome after e-SGB, etc.). These protocol violations will be shown in a listing. Subjects with puncture interruption and abandonment due to various reasons are considered to have failed e-SGB and will be excluded from any form of analysis. The decision to exclude a subject from the PP population will be made prior to breaking the blind. The primary outcome will be compared between groups using the Cochran–Mantel–Haenszel test. Postoperative cerebral hemodynamic changes measured at multiple planned time points will be compared using repeated-measures analysis of variance (ANOVA) with the Tukey–Kramer test. In addition, repeated-measures ANOVA will be utilized with a mixed-model approach (including time as a random effect), and the specific comparison of the change in each of those measurements between baseline and any specific time point will be tested using linear contrast. For other outcome data, categorical data will be analyzed by the *χ*^2^ test, and continuous data will be analyzed using Student’s *t* test or the Mann–Whitney *U* test. The rates of spasm, death, complications, and length of stay in the hospital and intensive care unit will be compared using Cox proportional hazards models. All analyses will be adjusted by age, sex, preoperative Hunt-Hess score, preoperative Fisher grade, and site of aneurysm. For the missing data, the last observation and the worst-case imputation scenarios will be used as the main interpolation method. A *p* value less than or equal to 0.05 (two-tailed alpha level) will be considered statistically significant.

### Discontinuation or withdrawal of study subjects

All subjects may voluntarily withdraw from the trial at any time for any reason, and the investigator may also discontinue the participation of any subject for a variety of reasons, primarily safety concerns or protocol violations.

### Data confidentiality management

All raw data will be collected through a paper case report form that is specially designed by researchers and placed in a dedicated locker. In addition, all researchers will follow the rules of professional confidentiality and must keep all personally identifiable and medical information of patients confidential. The paper clinical report form will be destroyed three years after the completion of the study. The electronic data will be encrypted and preserved after hiding the patient’s personal information, and access to the database will be restricted. All data confidentiality management shall be carried out by a designated data management team that is established by the principal investigator (PI) and independent statisticians. The PI will regularly inspect the content of the forms and the database to ensure accurate and timely data entry, follow-up, and recruitment progress as well as calculate withdrawal and loss-to-follow-up rates. At the end of this study, at least 20% of the clinical report forms will be randomly selected by the research department for integrity and authenticity review.

### Adverse event monitoring report and data monitoring committee

An adverse event (AE) is the development of an undesirable medical condition/complication or the deterioration of a preexisting medical condition following or during exposure to an intervention, whether considered causally related to the intervention. The expected harm/complications will be systematically collected by visiting each patient at each follow-up point, while the unexpected harm/complications will be collected through spontaneous reporting from the patients or physicians in charge. All study-related AEs will be recorded and closely monitored until they are resolved or stable or until it can be confirmed that the study treatment is not the cause of the events. The investigator will immediately report to the research department and advise the PI to determine the severity of the adverse event as soon as it occurs. In addition, it should be reported to the Ethics Committee (IRB) within 24 h, and the PI is responsible for reporting all AEs. The Data Monitoring Committee (DMC) will be responsible for monitoring clinical safety and reviewing all adverse events reported to the IRB to determine the risks and benefits of the study. The DMC is made up of specialists in anesthesiology, neuropathies, ethics, statistics, and methodology. If there were any severe adverse events (SAEs) associated with the study (grading criteria ≥ 3), the DMC was responsible for discussing whether to terminate the study (Table 2). In this study, all expected AE records will also be used as safety indicators to evaluate the safety of e-SGB, which is mainly completed by the DMC and reported in the final trial publications. If e-SGB significantly increases the risk of certain adverse events or is related to an unexpected SAE that deteriorates the patient’s condition, such as aneurysm rupture and rebleeding before clipping, we will emphasize this in the final results.

If the patient’s health is damaged due to participation in this study, we will be responsible for taking appropriate treatment measures. The sponsor, Beijing Tiantan Hospital, Capital Medical University, will bear the treatment expenses and pay corresponding economic compensation to the patients according to relevant national regulations.

### Ethics and dissemination

This study was approved by the Ethics Committee of Beijing Tiantan Hospital, Capital Medical University (Ethics No. KY 2021–023-02). In addition, it complies with the principles of the Helsinki Declaration, and the protocol (vol. 1.4; May 31, 2021) is written in accordance with the SPIRIT 2013 guidelines. The results of the study will be published in peer-reviewed journals, and the key findings will be presented at national or international conferences.

### Protocol amendment

Any decision to amend the trial protocol will be made by the PI. The PI will communicate with the research department before the implementation of the revised program if there are any modifications in the recruitment process of the study (such as changing the eligibility criteria, outcome indicators, statistical methods, etc.). Moreover, the PI shall obtain the approval of the Ethics Committee of Beijing Tiantan Hospital, Capital Medical University.

### Patients and public involvement

Patients and the public were not directly consulted during the formulation of the research questions or outcome measurements. None of the patients participated in the design, recruitment, or clinical implementation of this study. We will prepare a manuscript to present the trial results at the completion of the study. The final results of the study will also be disseminated to all study participants by their preferred method.

## Discussion

This is a prospective randomized controlled trial to explore the effectiveness and safety of e-SGB as a means of CVS prevention. DCI associated with CVS remains the main contributor to morbidity and mortality following aSAH. However, past efforts to address this issue have failed to make any considerable progress. Oral nimodipine has shown good curative effects but limited clinical application [5, 13]. Therefore, it is necessary to explore more feasible and effective treatments to reduce the incidence of CVS and DCI.

SGB is currently the most commonly used sympathetic block in medical practice. It has shown excellent curative effects on many intractable diseases, such as intractable angina, asthma, atopic dermatitis, hyperhidrosis, arrhythmias, hot flashes, and GI dysfunction. Recently, SGB was found to have a good therapeutic effect on posttraumatic stress disorder (PTSD) symptoms [37], which expanded its use to the field of neurology. Similarly, for vasospasm after aSAH, studies also found that SGB, as a remedial treatment, can alleviate progressive CVS and shows exciting potential [23, 29–33]. The expansion of applications of SGB to the whole body has been mirrored by expansion into pathophysiological conditions, which cannot be explained only by a direct lack of sympathetic innervation and consequent peripheral vasodilatation. As confirmed by numerous studies, it can modulate the immune response and diminish inflammation, which may be mediated by the generalized impact of sympathetic innervation on the immune system [21–29]. Therefore, similar to the “neuroprotection” of nimodipine, the potential protective effect of e-SGB after acute stress should not be ignored, as it may be the main mechanism for treating all of these diseases.

CVS usually occurs in 70% of patients between days 3 and 21 postbleeding, peaking on days 7 to 10 [5–8]. Increasing evidence supports that early neuroinflammatory reactions and EBI within the first 72 h are significant factors of CVS and DCI [5–8]. The initial contributor, hemoglobin, triggers an inflammatory cascade by sending signals to activate downstream signaling pathways. On the other hand, the acute increase in ICP due to bleeding and subsequent reduction in cerebral blood flow due to the functional and morphological deterioration of the cerebral arteries cause diffuse brain reactions and EBI. Neuroinflammation may catalyze EBI, which in turn increases neuroinflammation. In fact, this vicious cycle of inflammation probably contributes to almost all other mechanisms in the course of aSAH, including apoptotic or necrotic cell death, cortical spreading depression, blood–brain barrier disruption, and microthrombosis. A [38]. Therefore, blocking these inflammation-mediated processes in an early and timely manner may be more therapeutically effective. Current experimental studies observing anti-inflammatory treatments in the pathophysiology of CVS and DCI have shown promising potential, further suggesting the role of inflammation in vasospasm [38–41]. Toll-like receptor 4 (TLR4) is expressed in various cell types and exerts maximal inflammatory responses among the TLR family, which may be an important therapeutic target for patients with post-SAH brain injuries [12, 37]. A study observing improvement of neurological function in diabetic rats during ischemic stroke found that SGB can inhibit the Toll-like receptor 4/nuclear factor kappa B signaling pathway and reduce the inflammatory response in the plasma of rats [22]. Various proinflammatory mediators, such as cytokines, chemokines, and cellular targets, were also observed to be better regulated under SGB [25–27, 42–44]. In addition, compared with anti-inflammatory drugs, the effect of SGB on dilating arterial vessels may attenuate early hypoperfusion, which may be more conducive to alleviating various brain pathophysiologies, such as neuroinflammation, EBI, and cortical spreading depolarization, with fewer systemic adverse effects. All advances in these pathophysiologies involve deleterious vascular responses, which contribute to brain tissue early hypoperfusion. In summary, based on this rationale, we hypothesize that e-SGB may have an exciting potential role in preventing CVS.

The goal of reducing CVS is to reduce DCI and improve aSAH patients’ neurological outcome. Although the 90-day mRS score will be observed, it will not be used as the primary outcome, which is one of the shortcomings of this trial. However, as a prospective trial with the main purpose of exploring the safety and effectiveness of e-SGB, choosing an appropriate intermediate indicator can avoid a large sample cost demand. It seems imprudent to recruit vast numbers of patients when the effect is not clearly understood. Vasospasm, especially with clinical symptoms, is strongly correlated with devastating outcomes. On the other hand, a more effective e-SGB treatment protocol (such as continuous block and site selection) is currently unclear and needs to be explored to facilitate further research on improving long-term prognosis. TCD will be conducted to monitor continuous cerebral hemodynamics in this trial, with the aim of observing the effect of e-SGB on postoperative cerebral hemodynamics and exploring the timeliness of e-SGB. A sudden and rapid increase in blood flow velocity at a certain point is often indicative of disease progression. Second, the effect of a single e-SGB may be limited by the several-hour half-life of ropivacaine in this trial. However, in the PTSD study, KristineL et al. performed SGB on patients only twice (with a two-week interval), assessed at week 8 postintervention, and showed significant differences between the two groups [37]. Additionally, Christopher et al. found that the proportion of progressive CVS patients whose blood flow velocity improved 24 h after a single SGB was greater than 50%, of which 34.1% remained normal [31]. These results indicate that although the half-life of local anesthetics is only a few hours, their effect is longer. Early and timely intervention may be more important for the prevention of CVS and DCI. In this trial, e-SGB will be completed within the first 72 h of aSAH in a clinical setting. Finally, the measurement of cerebral blood flow velocity by TCD can only reflect cerebral perfusion laterally, and there may be false positives. Therefore, CTP will be routinely completed in all patients before discharge with the aim of assessing the improvement in cerebral perfusion following e-SGB in this trial.

In conclusion, successful completion of this trial and validation of its primary hypothesis would have implications for CVS prevention. If the result is positive, it may provide a new direction for the prevention and treatment of CVS and DCI.

## Trial status

This clinical study is currently in the recruitment phase. The study recruited the first patient on July 1, 2021, and the estimated study completion date will be December 30, 2023.

## Supplementary Information

**Additional file 1.** SPIRIT 2013 Checklist.

**Additional file 2.** Standardized Process and Indicator Definition.

## Data Availability

The datasets will be available from the primary investigator (Ruquan Han, Email: ruquan.han@ccmu.edu.cn) upon reasonable request after the publication of the study results.
