# Supplementary material for: Effect of early stellate ganglion block in cerebral vasospasm after aneurysmal subarachnoid hemorrhage (BLOCK-CVS): study protocol for a randomized controlled trial
Source: Trials. 2022 Nov 4;23:922. doi: 10.1186/s13063-022-06867-9 (PMC9636713; doi:10.1186/s13063-022-06867-9)
Supplement: Supplementary file 1 — Additional file 1. SPIRIT 2013 Checklist. [file 13063_2022_6867_MOESM1_ESM.doc]

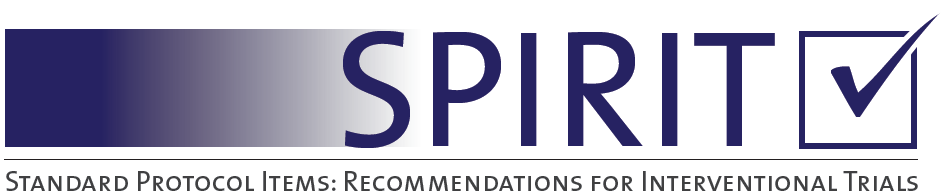


SPIRIT 2013 Checklist: Recommended items to address in a clinical trial protocol and related documents*

| Section/item | Item No | Description | Reported on NO. |
| --- | --- | --- | --- |
| **Administrative information** | | | **Page** |
| Title | 1 | Descriptive title identifying the study design, population, interventions, and,  if applicable, trial acronym | 1 |
| Trial registration | 2a | Trial identifier and registry name. If not yet registered, name of intended registry | 2-3 |
|  | 2b | All items from the World Health Organization Trial Registration Data Set | 2 |
| Protocol version | 3 | Date and version identifier | 2 |
| Funding | 4 | Sources and types of financial, material, and other support | 2 |
| Roles and responsibilities | 5a | Names, affiliations, and roles of protocol contributors | 1 |
|  | 5b | Name and contact information for the trial sponsor | 2 |
|  | 5c | Role of study sponsor and funders, if any, in study design; collection, management,  analysis, and interpretation of data; writing of the report; and the decision to submit  the report for publication, including whether they will have ultimate authority over  any of these activities | 2 |
|  | 5d | Composition, roles, and responsibilities of the coordinating centre, steering  committee, endpoint adjudication committee, data management team, and other  individuals or groups overseeing the trial, if applicable (see Item 21a for data  monitoring committee) | 12 |
| Introduction |  |  |  |
| Background and rationale | 6a | Description of research question and justification for undertaking the trial,  including summary of relevant studies (published and unpublished) examining  benefits and harms for each intervention | 4 |
|  | 6b | Explanation for choice of comparators | 4 |
| Objectives | 7 | Specific objectives or hypotheses | 5 |
| Trial design | 8 | Description of trial design including type of trial (eg, parallel group, crossover,  factorial, single group), allocation ratio, and framework (eg, superiority,  equivalence, noninferiority, exploratory) | 3,5 |
| Methods: Participants, interventions, and outcomes | | |  |
| Study setting | 9 | Description of study settings (eg, community clinic, academic hospital) and list of  countries where data will be collected. Reference to where list of study sites can  be obtained | 5 |
| Eligibility criteria | 10 | Inclusion and exclusion criteria for participants. If applicable, eligibility criteria for  study centres and individuals who will perform the interventions (eg, surgeons,  psychotherapists) | 5 |
| Interventions | 11a | Interventions for each group with sufficient detail to allow replication, including  how and when they will be administered | 6 |
|  | 11b | Criteria for discontinuing or modifying allocated interventions for a given trial  participant (eg, drug dose change in response to harms, participant request,  or improving/worsening disease) | 7,12, table2 |
|  | 11c | Strategies to improve adherence to intervention protocols, and any procedures  for monitoring adherence (eg, drug tablet return, laboratory tests) | Not applicable, intervention is a single treatment |
|  | 11d | Relevant concomitant care and interventions that are permitted or prohibited  during the trial | 8-9 |
| Outcomes | 12 | Primary, secondary, and other outcomes, including the specific measurement  variable (eg, systolic blood pressure), analysis metric (eg, change from baseline,  final value, time to event), method of aggregation (eg, median, proportion), and  time point for each outcome. Explanation of the clinical relevance of chosen  efficacy and harm outcomes is strongly recommended | 9-10 |
| Participant timeline | 13 | Time schedule of enrolment, interventions (including any run-ins and washouts),  assessments, and visits for participants. A schematic diagram is highly  recommended (see Figure) | 6,9, fig1, tab1 |
| Sample size | 14 | Estimated number of participants needed to achieve study objectives and how it  was determined, including clinical and statistical assumptions supporting any  sample size calculations | 10 |
| Recruitment | 15 | Strategies for achieving adequate participant enrolment to reach target sample size | No relevant strategy has been formulated in this study, but there are abundant cases in our centre (>300 cases per year. |
| **Methods: Assignment of interventions (for controlled trials)** | | |  |
| Allocation: |  |  |  |
| Sequence generation | 16a | Method of generating the allocation sequence (eg, computer-generated random  numbers), and list of any factors for stratification. To reduce predictability of a  random sequence, details of any planned restriction (eg, blocking) should be  provided in a separate document that is unavailable to those who enrol  participants or assign interventions | 6 |
| Allocation concealment mechanism | 16b | Mechanism of implementing the allocation sequence (eg, central telephone;  sequentially numbered, opaque, sealed envelopes), describing any steps to  conceal the sequence until interventions are assigned | 6 |
| Implementation | 16c | Who will generate the allocation sequence, who will enrol participants, and who  will assign participants to interventions | 6 |
| Blinding (masking) | 17a | Who will be blinded after assignment to interventions (eg, trial participants, care  providers, outcome assessors, data analysts), and how | 6 |
|  | 17b | If blinded, circumstances under which unblinding is permissible, and procedure  for revealing a participant’s allocated intervention during the trial | 6-7 |
| **Methods: Data collection, management, and analysis** | | |  |
| Data collection methods | 18a | Plans for assessment and collection of outcome, baseline, and other trial data,  including any related processes to promote data quality (eg, duplicate  measurements, training of assessors) and a description of study instruments  (eg, questionnaires, laboratory tests) along with their reliability and validity,  if known. Reference to where data collection forms can be found, if not in  the protocol | 9,11-12, fig1,tab1 |
|  | 18b | Plans to promote participant retention and complete follow-up, including list of  any outcome data to be collected for participants who discontinue or deviate  from intervention protocols | Not applicable, if the SGB intervention is failed, the case will be excluded |
| Data management | 19 | Plans for data entry, coding, security, and storage, including any related  processes to promote data quality (eg, double data entry; range checks for  data values). Reference to where details of data management procedures can  be found, if not in the protocol | 11-12 |
| Statistical methods | 20a | Statistical methods for analysing primary and secondary outcomes. Reference to  where other details of the statistical analysis plan can be found, if not in the protocol | 11 |
|  | 20b | Methods for any additional analyses (eg, subgroup and adjusted analyses) | 11 |
|  | 20c | Definition of analysis population relating to protocol non-adherence (eg, as  randomised analysis), and any statistical methods to handle missing data (eg,  multiple imputation) | 11 |
| **Methods: Monitoring** | | |  |
| Data monitoring | 21a | Composition of data monitoring committee (DMC); summary of its role and  reporting structure; statement of whether it is independent from the sponsor and  competing interests; and reference to where further details about its charter can  be found, if not in the protocol. Alternatively, an explanation of why a DMC is  not needed | 12 |
|  | 21b | Description of any interim analyses and stopping guidelines, including who will  have access to these interim results and make the final decision to terminate  the trial | 12 |
| Harms | 22 | Plans for collecting, assessing, reporting, and managing solicited and  spontaneously reported adverse events and other unintended effects of trial  interventions or trial conduct | 12 |
| Auditing | 23 | Frequency and procedures for auditing trial conduct, if any, and whether the  process will be independent from investigators and the sponsor | Not applicable, no auditing trial conduct |
| Ethics and dissemination | | |  |
| Research ethics approval | 24 | Plans for seeking research ethics committee/institutional review board  (REC/IRB) approval | 13 |
| Protocol amendments | 25 | Plans for communicating important protocol modifications (eg, changes to  eligibility criteria, outcomes, analyses) to relevant parties (eg, investigators,  REC/IRBs, trial participants, trial registries, journals, regulators) | 13 |
| Consent or assent | 26a | Who will obtain informed consent or assent from potential trial participants  or authorised surrogates, and how (see Item 32) | 6 |
|  | 26b | Additional consent provisions for collection and use of participant data and  biological specimens in ancillary studies, if applicable | Not applicable, these content was included in the patients'consent form |
| Confidentiality | 27 | How personal information about potential and enrolled participants will be  collected, shared, and maintained in order to protect confidentiality before,  during, and after the trial | 11-12 |
| Declaration of interests | 28 | Financial and other competing interests for principal investigators for the  overall trial and each study site | 2 |
| Access to data | 29 | Statement of who will have access to the final trial dataset, and disclosure of  contractual agreements that limit such access for investigators | 2 |
| Ancillary and post-trial care | 30 | Provisions, if any, for ancillary and post-trial care, and for compensation to  those who suffer harm from trial participation | 12 |
| Dissemination policy | 31a | Plans for investigators and sponsor to communicate trial results to participants,  healthcare professionals, the public, and other relevant groups (eg, via  publication, reporting in results databases, or other data sharing arrangements),  including any publication restrictions | 13 |
|  | 31b | Authorship eligibility guidelines and any intended use of professional writers | 2, no professional writers |
|  | 31c | Plans, if any, for granting public access to the full protocol, participant-level  dataset, and statistical code | 2,13 |
| Appendices |  |  |  |
| Informed consent materials | 32 | Model consent form and other related documentation given to participants  and authorised surrogates | supplement 2 |
| Biological specimens | 33 | Plans for collection, laboratory evaluation, and storage of biological specimens  for genetic or molecular analysis in the current trial and for future use in ancillary  studies, if applicable | Not applicable, no samples collected |

*It is strongly recommended that this checklist be read in conjunction with the SPIRIT 2013 Explanation & Elaboration for important clarification on the items. Amendments to the protocol should be tracked and dated. The SPIRIT checklist is copyrighted by the SPIRIT Group under the Creative Commons “[Attribution-NonCommercial-NoDerivs 3.0 Unported](http://www.creativecommons.org/licenses/by-nc-nd/3.0/)” license.
