# Supplementary material for: Effect of early stellate ganglion block in cerebral vasospasm after aneurysmal subarachnoid hemorrhage (BLOCK-CVS): study protocol for a randomized controlled trial
Source: Trials. 2022 Nov 4;23:922. doi: 10.1186/s13063-022-06867-9 (PMC9636713; doi:10.1186/s13063-022-06867-9)
Supplement: Supplementary file 2 — Additional file 2. Standardized Process and Indicator Definition. [file 13063_2022_6867_MOESM2_ESM.docx]

**
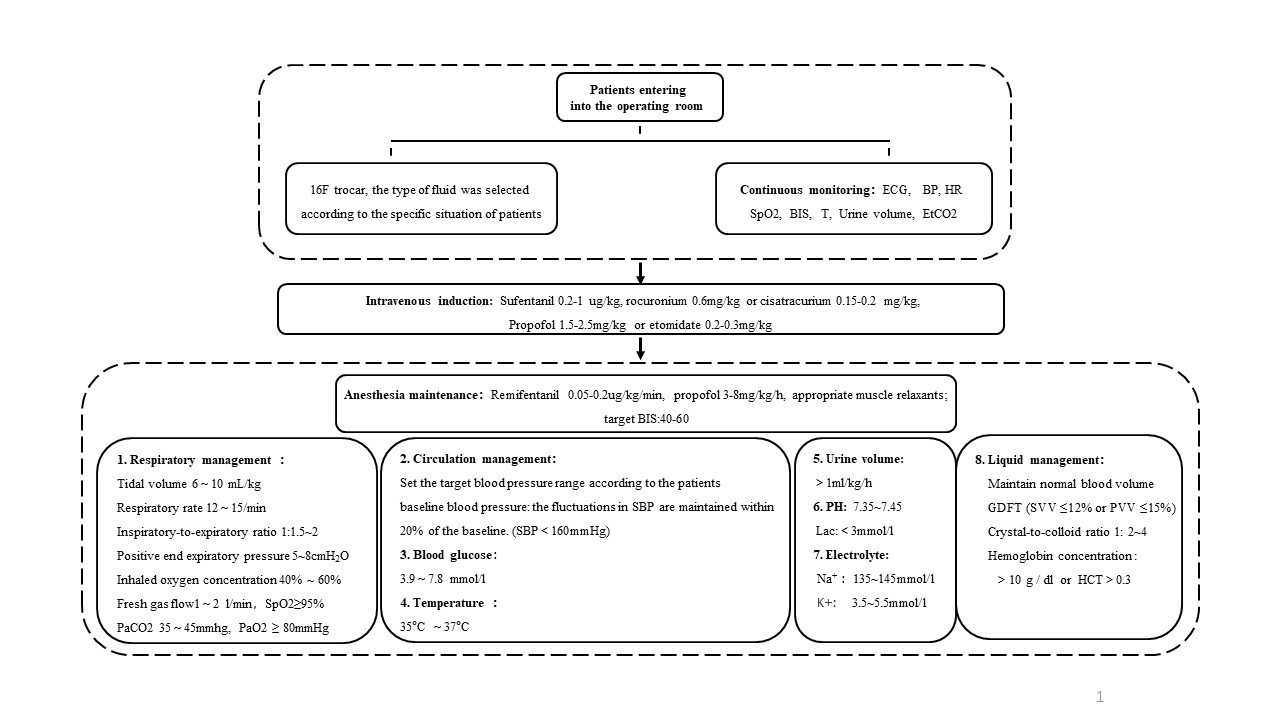
Supplement. Part 1. Standardized anesthesia management**

ECG, electrocardiogram; HR, heart rate; SpO2, pulse oxygen saturation; BP, blood pressure; BIS, bispectral index; T, temperature; EtCO2, end-tidal carbon dioxide;PaCO2, arterial partial pressure of carbon dioxide; PaO2, arterial partial pressure of oxygen; PH, [pH](javascript:;) [value](javascript:;); GDFT, Goal-directed fluid therapy; SVV, stroke volume variation; PVV, pulse pressure variation.

1. During the perioperative period, it is necessary to maintain normal blood volume. When the blood volume is sufficient (comprehensively judged by the attending anaesthesiologist, according to the loss and inflow of preoperative fluid, as well as SVV, PVV, HR, BP, UV, etc), vasoactive drugs can be used. Ephedrine, dopamine, deoxyepinephrine, or norepinephrine can be used as to maintain the blood pressure, and urapidil or nicardipine can be used as antihypertensive drugs.

2. The principle of fluid management is to maintain normovolemia and not actively use hypervolemia or hemodilution in the early stage. Patients with aSAH receive dehydration therapy (mannitol) to reduce intracranial pressure in the emergency room, so routinely given 1000ml crystalloid to replenish blood volume within the first 2 hours after entering the operating room. Whether or not to use colloid is determined by the attending anesthesiologist.

3. A short-term prophylactic antiepileptic therapy is permitted in our center. Sodium valproate 800mg is usually given at the end of surgery.

4. All patients are given tropisetron to prevent vomiting. Preoperative dysphoria can be sedated with midazolam as appropriate. Adequate analgesia should be given as much as possible during the perioperative period, and no analgesic pump is given after the operation. Postoperative pain was individually managed by the ICU unit. Try not to use respiratory stimulants (doxapram hydrochloride) or muscle relaxation antagonists (neostigmine).

5. Pay attention to the syndrome cerebral salt wasting，which is produced by excessive secretion of natriuretic peptides and causes hyponatremia from excessive natriuresis. In this setting, hydrocortisone can be used to help correcting the negative sodium balance.

**Supplement. Part 2. Patient management after surgery**

| **Intensive care monitoring** | Neurological examination: State of consciousness, verbal response, muscle force and muscle tension, neuro-pathological sign, and scale assessment: GCS, NIHSS, MMSE, RASS, CAM-ICU. The attending physician shall evaluate every day as far as possible.  Routine monitoring (applicable to all patients): ECG, BP, SpO2, T, UV.  Special monitoring:   - Brain imaging: CT within 24h and before discharge, CTA, and CTP before discharge (6-8days after operation). Whether an additional CT scan or CTA/CTP is determined by the attending physician based on the patient’s condition. - Chest X-ray: Once a week, if necessary, high-resolution CT can be added. - Lower extremity vein Doppler ultrasound: Once a week. - When necessary, cardiac ultrasound, pulmonary ultrasound and invasive blood pressure monitoring shall be conducted - TCD: The first three days after operation were monitored every day, and then every two days. - Laboratory examination: Routine blood test, coagulation function, C-reactive protein, liver function test, kidney function test, electrolyte, cardiac muscle enzymes, etc. Routine re-examination was performed once after operation, and then every 5 days. If the baseline or last examination result is abnormal, it shall be shortened to 2-3 days for re-examination. Blood-gas analysis once every two days is used to guide liquid treatment. Blood glucose was monitored daily. |
| --- | --- |
| **Airway management** | Keep the airway unobstructed and inhale oxygen if necessary. For patients with obvious respiratory dysfunction, endotracheal intubation or tracheotomy can be used to assist ventilation when necessary, and important indicators such as blood oxygen saturation can be monitored through blood gas analysis. Target control range: SpO2 ≥95%, PaCO2 35~45mmHg, PaO2≥80mmHg. |
| **Blood pressure management** | It is accepted that hypotension is not conducive to the prognosis of patients, but the optimal range of BP value is not understood. A higher level of BP may be more reasonable, so our center routinely maintains SBP at 140-220mmHg. We will not actively use induced hypertension to alleviate the ischemic symptoms. Only when the SBP of patients is lower than 140mmHg, we will use fluid therapy (10-15ml/kg) or norepinephrine to raise BP and keep it within the target range. Fluid therapy is the first choice and using norepinephrine only after the liquid treatment is ineffective. Colloidal liquid can be used to supplement blood volume and the ratio of crystal liquid to colloidal liquid is 2:1. |
| **Blood volume management** | Hypervolemia and hemodilution do not apply in our center, but maintenance of euvolemia and normal circulating blood volume is used to prevent DCI. According to the amount of dominant liquid in and out recorded in the past 24 hours, all patients receive a positive fluid balance >500 ml/day, which replenish daily non-dominant water loss. If the patient's body temperature increases, the non-dominant water loss will increase. For each increase of 1℃, the non-dominant water loss will increase by 300ml per day for additional supplement. If the patient has hypotension or hyperlactatemia, the competent physician can use liquid therapy as appropriate, 10ml/kg/d~15ml/kg/d to increase blood pressure or promote lactate elimination. |
| **Maintain intracranial pressure** | For patients with intracranial pressure directly monitored by intracranial catheterization, when ICP is higher than 20mmhg, it is necessary to consider actively reducing intracranial pressure. In patients without ICP monitoring, CT shows the edema, which is visible as areas of low density and loss of gray/white matter differentiation, on an unenhanced image or the obliteration of the cisterns and sulcal spaces, and at the same time, the patient is accompanied by symptoms such as headaches, nausea, vomiting, lethargy, altered mental status, confusion, coma, seizure or other manifestations. Similarly, they should also be actively treated with intracranial pressure reduction. Three percent NaCl or mannitol will be used to reduce intracranial pressure, preferably hypertonic saline due to its slight damage to the kidney. Continue to dynamically monitor the intracranial pressure of patients. If conservative treatment is ineffective or the condition worsens, decompressive craniectomy should be considered. |
| **Nimodipine treatment** | Intravenous continuous infusion of nimodipine 0.8-2.0 mg/h via an intravenous micropump is preferred at our center. Nimodipine treatment starts immediately when the patient enters the ICU after the operation. The initial dosage is 2mg / kg / h, but if the blood pressure cannot be maintained, the dosage of nimodipine will be reduced as appropriate. When the dose of nimodipine was lower than 0.8mg/kg/h, the intravenous infusion of nimodipine was stopped and replaced by low-dose and high-frequency oral nimodipine (30mg, q2h~q4h). At the same time, liquid therapy or vascular dosing will be started to maintain the blood pressure within the target range. |
| **Emergency treatment** | For patients with severe neurological deficit, which cannot be explained by rebleeding, hydrocephalus, fever, infection, electrolyte or metabolic disorders, surgical complications and etc, emergency DSA angiography will be performed, and intra-arterial vasodilators (Fasudil) or transluminal balloon angioplasty (TBA) will be given if necessary. Whether to initiate emergency treatment is decided by the attending neurologist. |
| **Other standardized treatment** | - **Temperature management:** The body temperature shall be controlled at ≤ 37 ℃. If the patient has fever, physical cooling is the first choice, followed by nonsteroidal drugs, such as Tylenol and ibuprofen. - **Sedation and analgesia:** The use of painkillers depends on the needs of patients. Flurbiprofen or low-dose continuous pumping of remifentanil can be selected. We do not routinely use sedation, but only when the patient has consciousness disorder, agitation or does not tolerate endotracheal intubation. Dexmedetomidine is the first choice, and midazolam can be added as appropriate. - **Antiepileptic therapy：**Routine use of valproate or levetiracetam if needed - **Heart rate:** Control the ventricular rate with esmolol if the patient's HR is greater than 130 bpm without fever. - **Electrolyte:** If the patient has hyponatremia, monitor the amount of 24-hour urinary sodium and supplement sodium as appropriate. At the same time, actively prevent hypernatremia. - **Nutrition support:** Ensure the energy supply of 25-30kcal / kg every day, and give patients enteral nutrition or parenteral nutrition if necessary - **Blood glucose:** Normal blood glucose, 3.9 mmol/L–7.8 mmol/L, was usually controlled after surgery, and insulin could be used when needed. - **Deep venous thrombosis:** Patients without DVT were given mechanical prophylaxis with lower extremity pumps, and patients with DVT were evaluated and treated with anticoagulation. |
| ECG, electrocardiogram; HR, heart rate; SpO2, pulse oxygen saturation; BP, blood pressure; SBP, systolic pressure; PaO2, arterial partial pressure of oxygen; PaCO2, arterial partial pressure of carbon dioxide; ETCO2, end-tidal carbon dioxide, UV, urine volume. | |

**Supplement. Part** 3. Definition of relevant indicators

**Primary outcome****:**

The incidence of symptomatic vasospasm within 14 days after aSAH, which is defined as new focal or global neurological dysfunction or a decrease in the Glasgow coma score by more than two points, and with angiographic vasospasm on TCD or CTA. Symptom cannot be explained by causes such as primary bleeding, rebleeding, hydrocephalus, fever, infection, electrolyte or metabolic disorders, and surgical complications. The nurse will check the patients every 4 hours. At 10:00 every day, the attending physician and nurse will evaluate whether the patient has symptomatic cerebral vasospasm in the last 24 hours and record it in the case report form.

**Secondary outcomes:**

- **TCD vasospasm:** The mean blood flow velocity (MFV) of the middle cerebral artery ≥ 120 cm/s or Lindegaard index (ratio of MFV of the middle cerebral artery to the internal carotid artery) ≥ 3. The MFV measured by TCD can reflect the degree of vascular stenosis to a certain extent: MFV at 120–140 cm/s indicates mild CVS with a stenosis less than 25%, MFV at 140–200 cm/s indicates moderate CVS whose stenosis is 25%–50%, MFV greater than 200 cm/s indicates severe CVS and a degree of lumen stenosis more than 50%.
- **CTA vasospasm:** Compared with the preoperative baseline, the corresponding vessel diameter narrowed by more than 30% or new segmental stenosis occurred, not related to atherosclerosis or mechanical artery stenosis caused by arterial clamps or coils. The diagnosis will be made by a neuroradiologist who blinded to the randomization sequence.
- **CTP:** This trial observed the difference in the proportion of abnormal CTP results between the two groups at discharge. The diagnosis will be made by a neuroradiologist who blinded to the randomization sequence.
- **New cerebral infarction** is defined only as the appearance of new low-density infarct shadow on CT image on the days 5-8 or at discharge compared with preoperation. Patients routinely undergoes a CT scan before surgery, within 24 hours and days 5-8 after the surgery, and before discharge. Whether an additional CT scan is required is determined by the attending physician based on the patient's condition. Postoperative CT within 24 h is used to assess whether early cerebral infarction or cerebral hemorrhage is caused by arterial stenosis due to surgery. The surgeon completes the assessment. But the new cerebral infarction is completed by an independent radiologist.
- **Continuous hemodynamic changes:** All blood flow indexes that can be obtained by TCD, including the changes in the mean blood flow velocity (mBFV), pulsatility index (PI), and resistance index (RI). Continuous monitoring will be conducted on the day before surgery (T0) and on the immediate (T1),1st (T2), 2nd (T3), 3rd (T4), 5th (T5) ,7th(T6), 9th(T7) days after operation.
- **MMSE and CAM-ICU:** Delirium will be documented using CAM-ICU during the first three days after surgery. Also, cognitive performance will be assessed at baseline, 7 days (or discharge) ,30 days, and 90days after surgery using mini-mental state examination (MMSE). To reduce the subjective influence, the scale evaluation was completed by an experienced psychometrician.
- **The modified Rankin scale (mRS)** : less than 2 points is defined as a good prognosis
- **Complications up to 90 days after randomization:** including myocardial infarction,  postoperative rebleeding, moderate and severe brain edema, pulmonary embolism, deep venous thrombosis.
- **SGB related complications:** Included infection and hematoma at the puncture site, block or injury of recurrent laryngeal/ phrenic and brachial plexus nerve, injury of vertebral artery, local anesthetic intoxation and complete spinal anesthesia. The diagnosis is completed by the competent anesthesiologist implementing interventions.
